# Supplementary material for: Plasma Total Antioxidant Capacity and Carbonylated Proteins Are Increased in Pregnant Women with Severe COVID-19
Source: Viruses. 2022 Mar 30;14(4):723. doi: 10.3390/v14040723 (PMC9025616; doi:10.3390/v14040723)
Supplement: Supplementary file 1 [file viruses-14-00723-s001.zip › viruses-1528046-supplementary.pdf]

Table S1. Effect of maternal characteristics and severity COVID-19 on oxidative stress markers.

	B	95 CI%	*p*-value	R2/(R2 adjusted)
**Malondialdehyde**				
Constant	36.7.	14.9 to 58.5	0.001	
Maternal age	−0.032	−0.434 to 0.370	0.874	0.0338 (−0.03529)
GA at diagnosis	−0.170	−0.528 to 0.188	0.344	
pBMI	−0.141	−0.527 to 0.245	0.468	
Severe COVID-19	−2.17	−6.60 to 2.26	0.330	
**Carbonylated proteins**				
Constant	12604	5077 to 20132	0.001	
Maternal age	−160	−299 to −21.3	0.025	0.1932 (0.1311)
GA at diagnosis	−1.38	−125 to 122	0.982	
pBMI	−65.5	−199 to 67.8	0.330	
Severe COVID-19	2032	504 to 3561	0.010	
**Total antioxidant capacity**				
Constant	43.8	−4.24 to 91.8	0.075	
Maternal age	0.052	−0.833 to 0.938	0.906	0.2240 (0.1643)
GA at diagnosis	0.071	−0.714 to 0.861	0.852	
pBMI	−0.213	−1.063 to 0.638	0.618	
Severe COVID-19	18.1	8.26 to 27.8	0.001	

Models included 54 COVID-19 pregnant women; non-severe COVID-19 (n = 40) and severe COVID-19 (n = 17); GA: Gestational age; pBMI: Pregestational body mass index.
